# Targeted Silencing of MART-1 Gene Expression by RNA Interference Enhances the Migration Ability of Uveal Melanoma Cells

**DOI:** 10.3390/ijms140715092

**Published:** 2013-07-19

**Authors:** Yidan Zhang, Renbing Jia, Jing Wang, Xiaofang Xu, Yuting Yao, Shengfan Ge, Xianqun Fan

**Affiliations:** 1 Department of Ophthalmology, Ninth People’s Hospital, Shanghai Jiao Tong University School of Medicine, Shanghai 200011, China; E-Mails: zhangyd513@126.com (Y.Z.); jrb6@sina.com (R.J.); wangjingyy1120@126.com (J.W.); xuxu0139@hotmail.com (X.X.); 2 Department of Clinical Laboratories, Ninth People’s Hospital, Shanghai Jiao Tong University School of Medicine, Shanghai 200011, China; E-Mail: yyt19870810@hotmail.com

**Keywords:** uveal melanoma, MART-1, NM23, migration ability

## Abstract

Uveal melanoma (UM) is the most common primary intraocular malignancy and the leading potentially fatal primary intraocular disease in adults. Melanoma antigen recognized by T-cells (MART-1) has been studied extensively as a clinically important diagnostic marker for melanoma, however, its biological function remains unclear. In the present study, the UM cell line SP6.5, which showed a high level of MART-1 expression, was subjected to small interfering RNA-mediated silencing of MART-1. Silencing of MART-1 expression increased the migration ability of SP6.5 cells and down-regulated the expression of the metastasis suppressor NM23. Our results suggest that MART-1 is a candidate target for the development of therapeutic strategies for UM and in particular for the suppression of metastasis associated with this malignancy.

## 1. Introduction

Uveal melanoma (UM) is the most common primary intraocular malignancy and the leading potentially fatal primary intraocular disease in adults. Although UM is uncommon in the general population, with an incidence of 5.3–10.9 cases per million per year [1,2], it is associated with high mortality and high metastatic potential. Approximately one-half of all UM patients will eventually die of this disease. In clinical practice, conventional treatments for UM such as chemotherapy, radiotherapy and surgical excision have shown limited efficacy. Other therapies that have been developed in the past decade include photocoagulation, transpupillary thermotherapy, charged particle irradiation, and immunotherapy. Immunotherapy, which has been extensively studied, is currently an important component of treatment regimens for malignant melanomas. The primary goals of the treatment of UM are preventing metastasis and saving lives, followed by the preservation of the eyeball and vision [3].

Melanoma antigen recognized by T cells (MART-1) is an antigen expressed by human melanoma cells that is recognized by cytotoxic T lymphocytes. Therefore, MART-1 is immunogenic in humans and has been widely used to induce anti-melanoma immunity in patients by means of several vaccination strategies. MART-1 is one of the melanocyte/melanoma differentiation antigens that are produced at high levels in early-stage melanosomes. It forms a complex with gp100 that is indispensable for melanosome structure and maturation [4,5]. MART-1 is a type III membrane protein with an apparent molecular mass of 22–24 kDa that localizes to the trans-Golgi network (TGN), the endoplasmic reticulum (ER), and melanosomes. MART-1 expression is restricted to melanocytes, melanomas, and the retinal pigment epithelium but it is absent from other tissues and tumors. Although it is a known biological marker that has been studied for many years, its function remains to be fully elucidated. In our previous study, Wang *et al.* reported the different expression patterns of MART-1 in UM cells. The results showed that the SP6.5 cell line had the highest expression levels of MART-1 among the four cell lines tested [6]. In this study, we selected the SP6.5 UM cell line based on its high level of MART-1 expression and used it to elucidate the biological function of the protein in UM by small interfering RNA (siRNA) mediated silencing of the MART-1 gene.

## 2. Results and Discussion

### 2.1. Silencing of MART-1 in SP6.5 Cells

**Figure 1:**
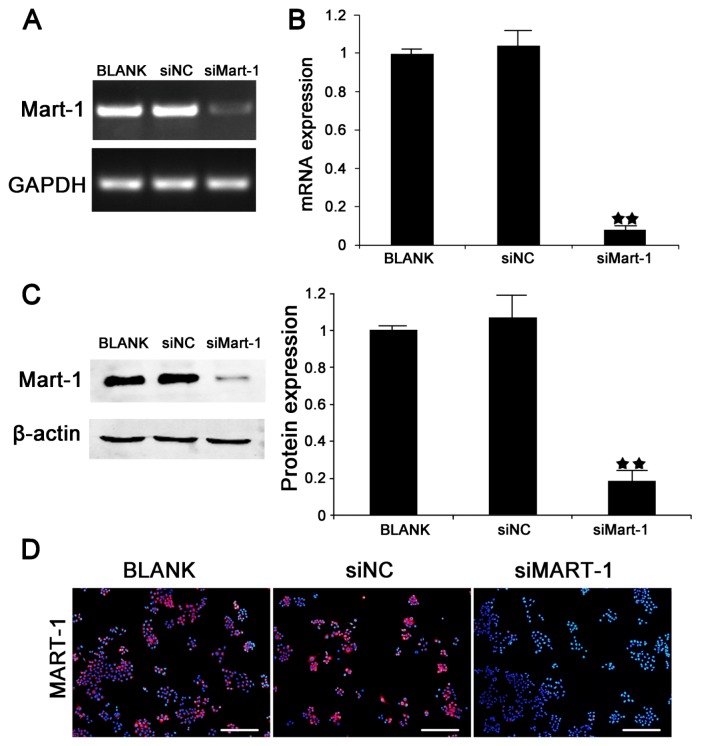
Targeted silencing of MART-1 gene expression by small interfering RNA. (**A**) Results of RT-PCR detection of MART-1 expression in SP6.5 cells transfected with BLANK, siNC, or siMart-1 for 24 h; (**B**) Quantitative real-time PCR analysis of MART-1 gene transcripts in SP6.5 cells. The experiment was performed after 24 h of siMart-1 (100 nmol/L) transfection. GAPDH was used as the internal control. ★★ *p* < 0.01 as compared with the negative control; (**C**) Western blot analysis of MART-1 protein levels in SP6.5 cells transfected with BLANK, siNC or siMart-1. The experiment was performed 48 h after siMart-1 transfection. Bands were scanned and values were normalized to those of the internal control β-actin; (**D**) Immunofluorescence detection of MART-1 in SP6.5 cells transfected with siMart-1, siNC or BLANK. Nuclei were stained with DAPI (blue), and MART-1 as visualized with goat anti-mouse IgG secondary antibody (red). Original magnification ×100. Scale was 100 μm in length. The results are representative of three independent experiments and of three replicates in each experiment.

SiRNA-mediated silencing of MART-1 expression was performed in SP6.5 cells to examine the role of MART-1 in UM. SP6.5 cells were transfected with siMart-1 and siNC (control siRNA), and the transfection efficiency was determined by PCR (Figure 1A), RT-PCR (Figure 1B) and western blotting (Figure 1C) and compared with untreated cells. The results showed that the transfection efficiency reached 80% or higher. MART-1 protein expression was then examined in transfected cells by immunofluorescence, which showed a strong staining pattern in the siNC (negative control) and BLANK (PBS blank control) transfected cells, whereas cells transfected with siMart-1 showed almost undetectable staining (Figure 1D).

### 2.2. Cell Cycle and Cell Proliferation in SP6.5 Cells with MART-1 Targeted Gene Silencing

**Figure 2:**
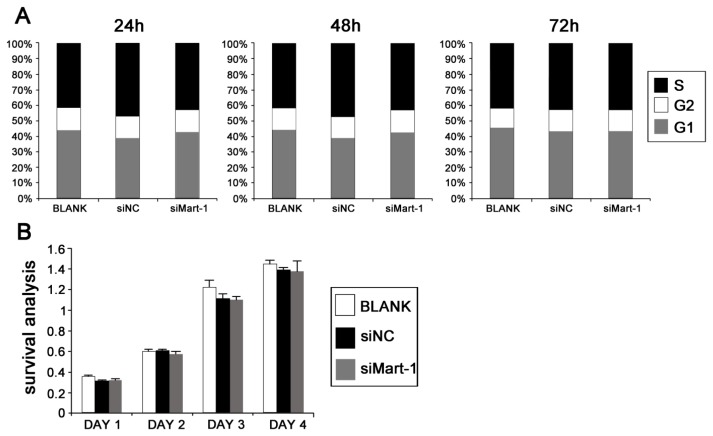
Effect of siMart-1 on cell cycle and cell proliferation. (**A**) Cell cycle was assessed by flow cytometry in cells transfected with BLANK, siNC and siMart-1 for 24, 48 and 72 h; (**B**) Cell proliferation of SP6.5 cells transfected with siMart-1 for 1, 2, 3 and 4 days was measured by MTT assay. Data are shown as the mean ± SD of three independent experiments.

To determine the role of MART-1 in cell cycle progression in UM cells, SP6.5 cells were analyzed by flow cytometry 24, 48 and 72 h after transfection with the different siRNAs. The results showed no significant differences in cell cycle pattern between target siRNA transfected cells and the controls (Figure 2A). In agreement with the flow cytometry results, assessment of cell proliferation by MTT assay showed similar cell viability in siMart-1 transfected cells and in the controls at 1, 2, 3 and 4 days (Figure 2B).

### 2.3. Effect of MART-1 Silencing on Cell Migration

**Figure 3:**
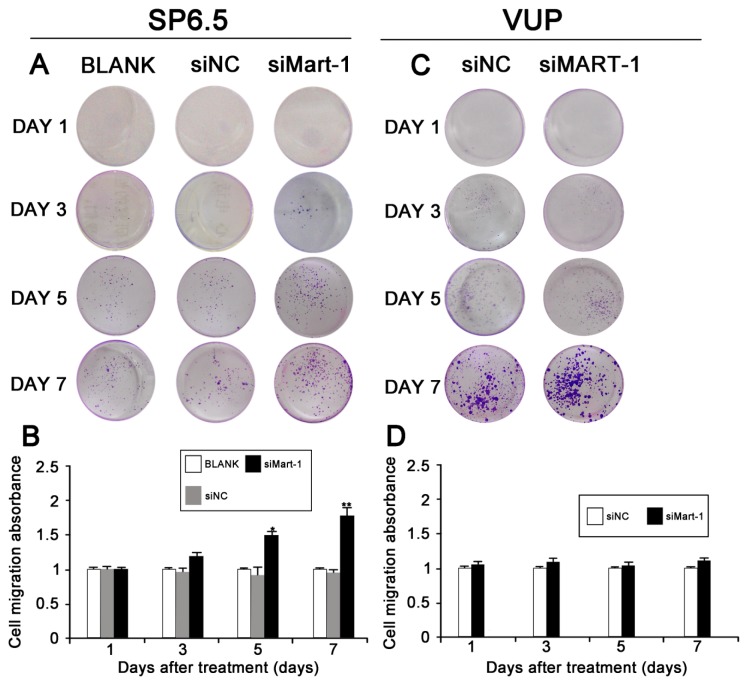
Effect of MART-1 silencing on cell migration of SP6.5 cells and VUP cells. (**A**) Image of the Transwell chamber assay showing SP6.5 cells treated as indicated for 1, 3, 5 and 7 days. The number of siMart-1 transfected cells invading through the filtration membrane was significantly higher than that of siNC and BLANK transfected cells at 5 and 7 days; (**B**) Cells were washed using 300 μL of 33% acetic acid and the absorbance of the wash liquid was determined with a microplate reader at 570 nm; (**C**) Image of the Transwell chamber assay showing VUP cells treated as indicated for 1, 3, 5 and 7 days. The number of siMart-1 transfected cells invading through the filtration membrane was the same as that of siNC cells; (**D**) Cells were washed using 300 μL of 33% acetic acid and the absorbance of the wash liquid was determined with a microplate reader at 570 nm. The results are representative of three independent experiments and of three replicates in each experiment. ★ *p* < 0.05 and ★★ *p* < 0.01, as compared with BLANK and siNC groups.

Next, we determined whether silencing of MART-1 expression had an effect on the migration of SP6.5 cells. SiRNA transfected cells were harvested at different times (1, 3, 5, and 7 days), stained with 0.1% crystal violet and photographed (Figure 3A). The result of the Transwell chamber assay indicated that the number of siMart-1 cells invading through the filtration membrane was higher than that of the controls, which was confirmed by measuring the absorbance of the wash liquid at 570 nm (Figure 3B). These results indicated that silencing MART-1 expression increased the migration ability of SP6.5 cells. As a negative control, we used VUP cells with low MART-1 expression levels; the results showed that there was no statistically significant difference between the migration ability of the siNC group compared to the siMART-1 group (Figure 3C,D).

### 2.4. MART-1 Silencing Decreases NM23 mRNA and Protein Levels

**Figure 4:**
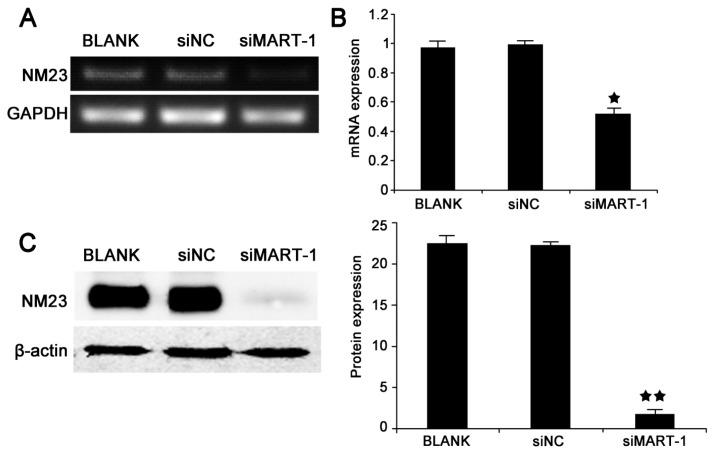
NM23 expression in SP6.5 uveal melanoma cells. (**A**) Results of PCR analysis of NM23 gene expression in SP6.5 cells transfected with siMart-1, siNC and BLANK for 24 h; (**B**) Quantitative real-time PCR analysis of NM23 gene expression in SP6.5 at 24 h after treatment; ★ *p* < 0.05, as compared with BLANK and siNC groups; (**C**) Western blot analysis of NM23 protein expression in SP6.5 cells transfected with siMart-1, siNC and BLANK at 48 h. Bands were scanned and normalized to the internal control β-actin. The results are representative of three independent experiments and of three replicates in each experiment. ★★ *p* < 0.01, as compared with BLANK and siNC groups.

Changes in cell migration induced by MART-1 gene silencing led us to examine the NM23 gene. To determine the effect of MART-1 silencing on the expression of the metastasis suppressor NM23 at the mRNA and protein levels, SP6.5 cells were analyzed by reverse transcriptase PCR, (Figure 4A), quantitative real time PCR (Figure 4B), and western blotting (Figure 4C) after 24 h of siRNA transfection. The results showed significant differences in NM23 mRNA (*p* < 0.01) and protein (*p* < 0.01) levels between siMart-1 and control cells, and indicated that silencing of MART-1 expression significantly down-regulated NM23 expression.

### 2.5. Effect of MART-1 Silencing on BAP1 mRNA and Protein Levels

**Figure 5:**
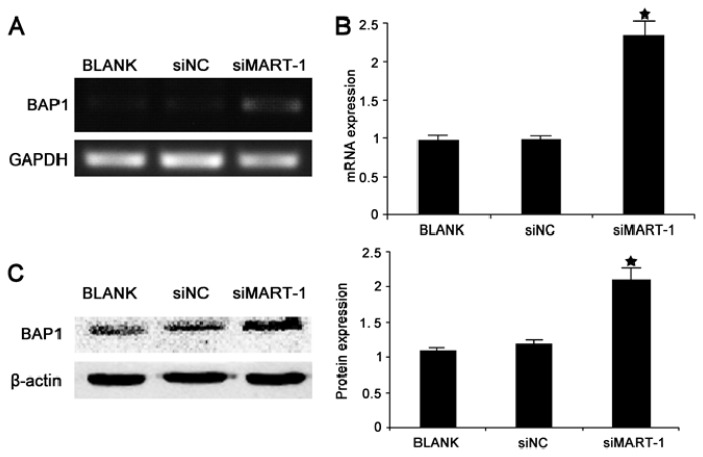
BAP1 expression in SP6.5 uveal melanoma cells. (**A**) PCR analysis of BAP1 expression in SP6.5 cells transfected with siMart-1, siNC and BLANK for 24 h; (**B**) Quantitative real-time PCR analysis of BAP1 gene expression in SP6.5 cells transfected with siMart-1, siNC and BLANK for 24 h; (**C**) Western blot analysis of BAP1 protein expression at 48 h in SP6.5 cells transfected with BLANK, siNC and siMart-1. Bar, average band density of quantified BAP1 protein following normalization by the internal control β-actin. ★ *p* <0.05, as compared with BLANK and siNC groups.

Analysis of the expression of the cancer suppressor gene BAP1 was performed as described above using PCR and RT-PCR 24 h after transfection of SP6.5 cells with siMart-1. The results showed statistically significantly higher BAP1 expression in siMart-1 cells compared to control cells (*p* < 0.05) (Figure 5A,B). To confirm the effect of MART-1 silencing at the protein level, cells were analyzed by western blotting 48 h after transfection with siMart-1 or control siRNA, which showed statistically significant increased BAP1 protein levels (*p* < 0.05) (Figure 5C).

### 2.6. Discussion

Uveal melanoma (UM), is the most common primary intraocular tumor in adults. There are two main challenges in the design of treatment strategies for UM, drug resistance and a high rate of metastasis. Approximately one half of the patients with UM die from metastases [7]. Therefore, the successful treatment of patients with UM requires finding solutions to these two problems. MART-1, an important determinant of melanosome maturation and development, is a type III transmembrane protein that is present in 75%–100% of primary and metastatic tumors of melanocytic origin and is expressed in normal melanocytes. Although MART-1 has been recognized as a useful melanoma marker, its biological function remains unclear. In the present study, to gain a better understanding of the biological function of MART-1, we used targeted silencing of the MART-1 gene in UM cell lines. RNA interference is a sequence specific strategy for gene therapy that is based on the modulation of the expression of target genes [8]. It has been used extensively as an experimental tool to dissect cellular pathways and gene function. Therefore, we used small interfering RNA targeting to silence the expression of the MART-1 gene in SP6.5 cells. The results showed the efficient inhibition of MART-1 expression at the mRNA and protein levels (Figure 1). To further investigate the biological functions of MART-1, we performed different experiments to examine the effects of MART-1 silencing on cell cycle progression and cell proliferation (Figure 2). The results showed that interference with MART-1 had no effect on the cell cycle and cell proliferation. Mariana *et al.* reported that melanoma cells expressing and not expressing MART-1 were equally proliferative, which is in agreement with our results [9].

Metastasis is a critical factor in the prognosis of cancer patients and a major contributor to mortality. Because UM is highly metastatic disease, we examined the effect of MART-1 silencing on cell migration by the Transwell chamber assay, using the differently treated cells at multiple time points. Our results showed that silencing MART-1 expression significantly increased the migration ability of SP6.5 cells (Figure 3A,B). We used VUP cells with low MART-1 expression as a negative control, and the results showed no statistically significant differences in the migration ability between the siNC group and the siMART-1 group. In our experiment, the migration ability of the VUP cell group was stronger than that of the SP6.5 BLANK group and siMART-1 group, especially on the 7th day. From the results, we predicted that MART-1 might be a metastasis-associated gene of UM.

Tumor metastasis is an essential aspect of uveal melanoma progression. There are many factors that can affect UM cell migration. The pharmacological NF-κB inhibitor, BAY11-7082, has been reported to induce cell apoptosis and inhibit the migration of human uveal melanoma cells [10]. High PTP4A3 phosphatase expression correlates with metastatic risk in uveal melanoma patients [11]. In addition, a direct link between the central PI3K/Akt pathway and uveal melanoma migration important for therapeutic intervention has been reported in hepatic metastasis [12]. Florenes *et al.* found low levels of NM23 mRNA in metastatic malignant melanomas [13]. Furthermore, a study showed that NM23 gene expression in bioptic tissue might represent an extremely useful prognostic tool for metastatic progression of uveal melanomas [14]. In our study, because the migration ability in the siMART-1 group was increased, we examined the expression of the metastasis suppressor gene NM23.

NM23, the first metastasis suppressor gene, possesses three enzymatic activities *in vitro*: a nucleoside diphosphate kinase (NDPK) activity, a protein histidine kinase activity, and a more recently characterized 3′-5′ exonuclease activity [15,16]. Luo *et al.* found that the lung cancer cell lines with stable NM23-H1 gene silencing were successfully established and their invasiveness was greatly increased after NM23-H1 gene knockdown [17]. Increasing evidence suggests that NM23 may act as a metastasis suppressor gene in certain forms of cancer. Our results showed statistically significant differences in NM23 protein and gene expression between cells transfected with siMart-1 and control cells (Figure 4). There are two possible explanations for this result: the MART-1 protein may be related to the migration ability of UM cells or MART-1 may be involved in the cellular pathway of the NM23 gene. The 3′-5′ exonuclease activity of NM23-H1 is necessary for its metastasis suppressor function, although the three enzymatic activities of the molecule act cooperatively in the suppression of the metastatic process [15]. MART-1 may also be involved in these activities, although further study is required to clarify its exact involvement.

BAP1 (BRCA1 associated protein-1) is a deubiquitinating enzyme that was first identified as a BRCA1 binding protein in a yeast two hybrid screen and subsequently shown to have a mild synergistic effect on BRCA1-mediated growth suppression [18,19]. The BAP1 gene has been suggested to be a tumor suppressor with a role in cell proliferation and growth inhibition [20,21]. Harbour *et al.* reported that the BAP1 gene was frequently mutated in metastasizing UMs [22]. We used full genome sequencing of the SP6.5 cell line to examine BAP1; the results showed that the SP6.5 cell line did not contain a BAP1 gene mutation (data not shown). In the present study, silencing of MART-1 expression increased the levels of the BAP1 protein to a certain extent (Figure 5), suggesting that BAP1 may be associated with a transitory increase in a short-term stress.

In summary, targeted silencing of MART-1 expression increased the migration of UM cells and decreased NM23 gene expression. These results suggest that MART-1 may be a metastasis-associated gene in UM. Future studies should focus on elucidating the underlying mechanisms of MART-1 activity; the overexpression experiment in the cell lines with low MART-1 expression is a direction for future research and examining its potential role as a molecular target for the treatment of UM.

## 3. Experimental Section

### 3.1. Cell Lines and Cell Culture

The human UM cell lines SP6.5, VUP, were kindly provided by Marshall [23]. These UM cells were composed of a mixture of spindle and epithelioid cells, and all UM cells analyzed were derived from primary tumors of choroidal and ciliary bodies. The VUP cell lines were mainly composed of epithelioid cells, while the SP6.5 cell line was of a mixed spindle-epithelioid cell type [24]. UM cells were maintained in Dulbecco’s Modified Essential Medium (DMEM) (Gibco, Carlsbad, CA, USA) with 10% fetal bovine serum, 100 U/mL penicillin and 100 mg/mL streptomycin (Mediatech, Herndon, VA, USA); Cells were incubated at 37 °C in a 5% CO_2_ atmosphere [25].

### 3.2. MART-1 siRNA Oligonucleotides

MART-1 siRNA oligonucleotides (siMart-1: 5′ TCCGCTAGCAGTACTAATCAT 3′) [26] and control siRNA (siNC: 5′ UUCUCCGAACGUGUCACGUTT 3′) were selected based on published synthetic siRNA sequences (Shanghai Genepharma, Shanghai, China).

### 3.3. SiRNA Transfection

When cells reached 70% confluency, they were trypsinized, and were seeded in 6-well plates at a density of 2 × 10^5^ cells/well. After 24 h of culture, cells were transfected with 100 nM/L of each siRNA using the Dharma-FECT transfection reagent (Thermo Fisher Scientific, Waltham, MA, USA) according to the manufacturer’s instructions. After 12–16 h, the transfection medium was replaced by fresh medium. The transfection efficiency was typically greater than 80%. MART-1 silencing efficiency was determined by quantitative real-time PCR, western blotting and immunocytochemistry.

### 3.4. RNA Extraction and Reverse Transcription-PCR Analysis

Total RNA was isolated from siRNA and control-transfected UM cells using the Trizol reagent (Invitrogen) following the protocol provided by the manufacturer. The cDNA was used to amplify the MART-1 fragments. For normalization of RNA, the housekeeping gene GAPDH was also amplified from each sample. The primer sequences used were: MART-1 forward, 5′ CACGGCCACTCTTACACCAC 3′; reverse, 5′ GGAGCATTGGGAACCACAGG 3′, 254bp; and GAPDH forward, 5′ GGATTTGGTGGTATTGGG 3′; reverse, 5′ GGAAGATGGTGATGGGATT 3′, 205 bp.

### 3.5. Quantitative Real-Time PCR

Quantitative real-time-PCR was performed using an ABI Prism 7500 Sequence Detection System (Applied Biosystems, Foster City, CA, USA) and the SYBR Premix Ex Taq (Takara, Tokyo, Japan). The cDNA was synthesized from 1 mg of total RNA using the PrimeScript RT reagent kit (Takara). Specific primer sequences were as follows: Mart-1 forward, 5′ CACGGCCACTCTTACACCAC 3′; reverse, 5′ GGAGCATTGGGAACCACAGG 3′; GAPDH forward, 5′ CGGATTTGGTGGTATTGGG 3′; reverse, 5′ GGAAGATGGTGATGGGATT 3′. PCR cycle parameters were the same for both primers used and consisted of 30 cycles of denaturation at 94 °C for 30 s, annealing at 60 °C for 40 s and extension at 72 °C for 50 s. The amplified products were analyzed by electrophoresis on 2% agarose gels, stained with ethidium bromide and visualized under UV light [6].

### 3.6. Western Blot Analysis

MART-1 protein expression in UM and ARPE-19 cells was examined by western blotting as previously described [27]. The cells were harvested, washed in cold phosphate-buffered saline (PBS) and lysed with lysis buffer (Fermentas). Protein was extracted and protein concentration was determined using the BCA protein assay kit (Thermo, Rockford, IL, USA). Equal amounts of protein were separated by sodium dodecyl sulfate-polyacrylamide gel electrophoresis on 12% (*w*/*v*) polyacrylamide gels, and transferred to polyvinylidene fluoride membranes. Membranes were incubated with the indicated primary antibodies followed by incubation with the corresponding secondary antibody conjugated to a fluorescent tag. Finally, the bands were visualized using the Odyssey Infrared Imaging System (LI-COR, Lincoln, NE, USA). The antibodies used were MART-1 (Abcam, Hong Kong), BAP1 (Santa Cruz Biotechnology, Santa Cruz, CA, USA), NM23 (Abcam, Hong Kong) and anti-β-actin (Sigma, St. Louis, MO, USA).

### 3.7. Immunocytochemistry

Cells were collected, attached to glass slides, fixed by 4% paraformaldehyde for 30 min and incubated with 0.1% Triton X-100 and 5% dimethylsulfoxide in PBS for 10 min. The cells were subsequently blocked with normal goat serum (Invitrogen) at 37 °C for 30 min. After rinsing with cold PBS twice, cells were incubated with the mouse monoclonal anti-MART-1 antibody M2-7C10 at 37 °C for 1 h. Subsequently, the cells were gently rinsed twice in cold PBS and incubated with goat anti-mouse IgG secondary antibody (1:1000 dilution in PBS/0.5% BSA; Invitrogen) and DAPI (4′,6-diamidino-2-phenylindole, 1:1000 dilution in PBS/0.5% BSA) at 37 °C for 30 min in the dark. The cell smears were finally placed on coverslips and photographed under a fluorescence microscope at 490–520 nm.

### 3.8. Cell Cycle

Cells were seeded at 2 × 10^5^ cells per well in flat-bottomed 6-well plates. Cells were harvested after 24, 48 and 72 h of treatment with BLANK, siNC, and siMart-1, washed twice with PBS and stained with 10 μg/mL propidium iodide. Cell cycle distribution was determined by flow cytometry using FACS Calibur (Becton-Dickinson and Company, Franklin Lakes, NJ, USA) and analyzed using CELL Quest software [28].

### 3.9. MTT Assay

The 3-(4, 5-dimethylthiazol-2-yl)-2, 5-diphenyl-2H-tetrazolium bromide (MTT) assay was performed to assess the effect of siMart-1 on SP6.5 cell proliferation. SP6.5 cells were seeded in 96-well plates at a density of 1 × 10^5^ cells/well. At the end of the incubation period, 20 μL of 5 mg/mL MTT (Sigma) in PBS was added to each well. Each experiment was repeated three times. Absorbance was measured after incubation for a further 4 h at 37 °C with a solution of MTT (0.2 mg/mL) containing 12.5 μM dimethyl sulfoxide (DMSO). Absorbance was measured on a microplate spectrophotometer at a wavelength of 490 nm [28].

### 3.10. Migration Assay

The migration assay was based on the migration of UM cells seeded in an upper chamber through a membrane of 8μm pore size (Millipore, Schwalbach, Germany). Cells were seeded into 6-well plates at a density of 2 × 10^5^ cells/well, cultured for 18–24 h and then transfected with 50 nM of each siRNA using Dharma-FECT transfection reagent. After 6–12 h the medium was replaced with fresh medium. At 24 h after transfection, cells were detached and collected. The upper chamber was seeded with 200 μL 1 × 10^5^ cells of the differently treated cells in DMEM plus 1% FBS and the lower chamber contained 1ML of DMEM plus 20% FBS was added to each well of the 24-well plate. Incubation at 37 °C, the lower chamber was fixed with 100% methanol and stained with 0.1% crystal violet. The crystal violet was washed from the migrated cells using 300 μL of 33% acetic acid, and the absorbance of the collected liquid was measured with a microplate reader at 570 nm [10]. Computational cells mobility: cell migration rate = the experimental group (BLANK/siNC/siMART-1) migration cells absorbance/BLANK control group absorbance.

## 4. Conclusions

In conclusion, our findings show that targeted silencing of MART-1 expression increased the migration of UM cells and decreased NM23 gene expression, suggesting that MART-1 may be a metastasis suppressor gene of UM. However the underlying mechanisms still need to be elucidated in order to enhance the efficacy of gene therapy.
